# Prevalence and Trends in Diagnosed ADHD Among US Children and Adolescents, 2017-2022

**DOI:** 10.1001/jamanetworkopen.2023.36872

**Published:** 2023-10-04

**Authors:** Yanmei Li, Xiaofang Yan, Qishan Li, Qian Li, Guifeng Xu, Jinhua Lu, Wenhan Yang

**Affiliations:** 1 Department of Child and Adolescent Health, School of Public Health, Guangdong Pharmaceutical University, Guangzhou, Guangdong Province, China; 2 Department of Pediatrics, The First Affiliated Hospital, University of Science and Technology of China, Hefei, Anhui Province, China; 3 Division of Birth Cohort Study, Guangzhou Women and Children’s Medical Center, Guangzhou Medical University, Guangzhou, Guangdong Province, China

## Abstract

This cross-sectional study estimates prevalence and trends of ADHD diagnosis among US children and adolescents from 2017 to 2022.

## Introduction

Attention-deficit hyperactivity disorder (ADHD) is the most common childhood-onset neurodevelopmental disorder, characterized by persistent and impairing inattention, hyperactivity, and impulsivity, with a high prevalence among US children and considerable implications for individuals and families.^1,2^ Studies have found that prevalence of ADHD increased from 1997 to 2016 in US children. An analysis of the National Health Interview Survey (NHIS)^2^ reported that the prevalence of ADHD among children increased from 6.1% in 1997 to 1998 to 10.2% in 2015 to 2016. Similarly, the National Survey of Children’s Health showed a 42.0% increase from 2003 to 2011.^3^ However, the latest prevalence has not been updated in the past 6 years. The aim of this study was to estimate the prevalence and trends of ADHD among children and adolescents in the US in 2017 to 2022.

## Methods

Data for this cross-sectional study were obtained from the NHIS, 2017 to 2022, a nationally representative cross-sectional survey using multistage, stratified sampling.^4^ Information about ADHD diagnosed by a physician or other health professional was reported by a parent or guardian. The final sample child and adolescent response rate was 45.8% to 60.6% from 2017 to 2022. The Guangdong Pharmaceutical University academic review board deemed the study exempt from review because of the use of deidentified data. The NHIS has been approved by the National Center for Health Statistics ethics review board, and all respondents provided verbal informed consent before participation. The study followed the STROBE reporting guideline.

The weighted prevalence of ADHD among individuals in the US aged 4 to 17 years from 2017 to 2022 was calculated based on the complex sampling design following NHIS statistical guidelines.^5^ *P* values for overall differences across strata were calculated using χ^2^ tests. Trends in prevalence over time were tested using a weighted logistic regression model, which adjusted for age, sex, race and ethnicity, family educational level, and family income-to-poverty ratio. Race and ethnicity were self-reported in the survey and were assessed to gather demographic information for health care research and investigate health disparities. Statistical analyses were conducted using survey procedures in SAS statistical software version 9.4 (SAS Institute). A 2-sided *P* < .05 was considered statistically significant.

## Results

A total of 37 609 individuals aged 4 to 17 years (18 185 female [48.35%] and 19 424 male [51.65%]; 9030 Hispanic [24.01%], 4119 non-Hispanic Black [10.95%], 19 816 non-Hispanic White [52.69%], and 4644 non-Hispanic other race [12.35%]) were included. Among them, 4098 children and adolescents (10.90%) were reported to have ever been diagnosed with ADHD. The weighted prevalence of ADHD was 10.20% (95% CI, 9.54%-10.87%) in 2017 to 2018, 10.08% (95% CI, 9.33%-10.83%) in 2019 to 2020, and 10.47% (95% CI, 9.81%-11.13%) in 2021 to 2022. There were significant differences in prevalence by age, sex, race and ethnicity, and family income-to-poverty ratio (Table).

**Table.** Prevalence of Diagnosed ADHD Among Children and Adolescents, 2017-2022

Characteristic	2017-2018			2019-2020			2021-2022			
	Participants with ADHD		*P* value^c^	Participants with ADHD		*P* value^c^	Participants with ADHD		*P* value^c^	*P* for trend^d^
	No./total^a^	% (95% CI)^b^		No./total^a^	% (95% CI)^b^		No./total^a^	% (95% CI)^b^		
Overall	1459/13 434	10.20 (9.54-10.87)	NA	1256/11 785	10.08 (9.33-10.83)	NA	1383/12 390	10.47 (9.81-11.13)	NA	.93
Age, y										
4-11	582/7122	7.85 (7.09-8.61)	<.001	475/5893	7.66 (6.71-8.61)	<.001	514/6427	7.54 (6.79-8.30)	<.001	.46
12-17	877/6312	13.36 (12.32-14.39)		781/5892	13.15 (11.99-14.30)		869/5963	14.16 (13.09-15.23)		.39
Sex										
Male	1020/7001	13.71 (12.69-14.74)	<.001	856/6048	13.12 (12.07-14.16)	<.001	903/6375	13.42 (12.39-14.45)	<.001	.46
Female	439/6433	6.59 (5.87-7.32)		400/5737	6.92 (6.02-7.81)		480/6015	7.38 (6.68-8.09)		.19
Race and ethnicity^e^										
Hispanic	230/3043	7.46 (6.29-8.63)	<.001	230/2780	7.86 (6.61-9.11)	<.001	263/3207	8.07 (6.91-9.22)	<.001	.89
Non-Hispanic Black	196/1544	11.96 (9.90-14.01)		131/1299	9.51 (7.42-11.59)		143/1276	10.76 (8.58-12.94)		.30
Non-Hispanic White	911/7308	11.57 (10.70-12.44)		794/6323	11.81 (10.73-12.89)		869/6185	12.43 (11.52-13.33)		.45
Other^f^	122/1539	7.88 (6.18-9.57)		101/1383	7.41 (5.65-9.16)		108/1722	6.59 (5.25-7.94)		.12
Highest educational level of family members										
<High school	145/1196	10.98 (8.96-13.00)	.39	81/763	8.96 (6.26-11.67)	.47	86/760	10.39 (7.85-12.93)	.50	.82
High school	224/1913	10.96 (9.27-12.66)		202/1753	10.97 (9.10-12.85)		227/1899	11.36 (9.65-13.07)		.85
≥College	1089/10 308	9.96 (9.21-10.72)		972/9258	10.01 (9.17-10.86)		1067/9715	10.29 (9.57-11.01)		.95
Missing	1/17	4.68 (<0.01-13.97)	NA	1/11	15.03 (<0.0141.55)	NA	3/16	13.43 (<0.01-28.64)	NA	NA
Family income-to-poverty ratio^g^										
<1.00	224/1484	12.98 (11.03-14.94)	<.001	216/1473	13.71 (11.45-15.97)	<.001	218/1475	13.27 (11.13-15.40)	.002	.98
1.00-1.99	281/2254	11.89 (10.36-13.41)		294/2421	11.10 (9.55-12.65)		283/2539	10.59 (9.27-11.90)		.14
2.00-3.99	363/3486	9.39 (8.17-10.61)		360/3687	8.49 (7.39-9.59)		377/3713	9.56 (8.47-10.65)		.42
≥4.00	409/4177	9.43 (8.35-10.52)		386/4204	8.97 (7.87-10.08)		505/4663	9.90 (8.87-10.92)		.44
Missing	182/2033	8.54 (7.09-10.00)	NA	NA	NA	NA	NA	NA	NA	NA

Abbreviations: ADHD, attention-deficit hyperactivity disorder; NA, not applicable.

^a^ The number of participants was unweighted.

^b^ Prevalence of ADHD was weighted.

^c^ *P* value for overall differences in prevalence by stratum.

^d^ *P* for trend was calculated using a weighted logistic regression model, which included survey cycle as a continuous variable and was adjusted for age, sex, race and ethnicity, family educational level, and family income.

^e^ Race and ethnicity were self-reported and classified based on the 1997 Office of Management and Budget standards. Categories in the survey were Hispanic (multiple Hispanic; Puerto Rico; Mexican; Mexican American; Dominican [Republic]; Central or South American; other Latin American, type not specified; other Spanish; Hispanic/Latino/Spanish, nonspecific type; Hispanic/Latino/Spanish, type refused; Hispanic/Latino/Spanish, type not ascertained), not Hispanic/Spanish origin, non-Hispanic Black (non-Hispanic Black/African American only), non-Hispanic White (non-Hispanic White only), and other.

^f^ Other races and ethnicities included non-Hispanic American Indian or Alaska Native (individual only), non-Hispanic American Indian or Alaska Native and any other group, non-Hispanic Asian (individual only), and other single and multiple races or declined to respond, no response, or unknown.

^g^ The ratio is the total family income divided by the poverty threshold.

Prevalence had no significant change annually (10.00% [95% CI, 9.11%-10.90%] in 2017; 10.80% [95% CI, 9.83%-11.76%] in 2022; *P* for trend = .31). All subgroups evaluated also showed no significant change in prevalence from 2017 to 2022 (Figure).

**Figure.**
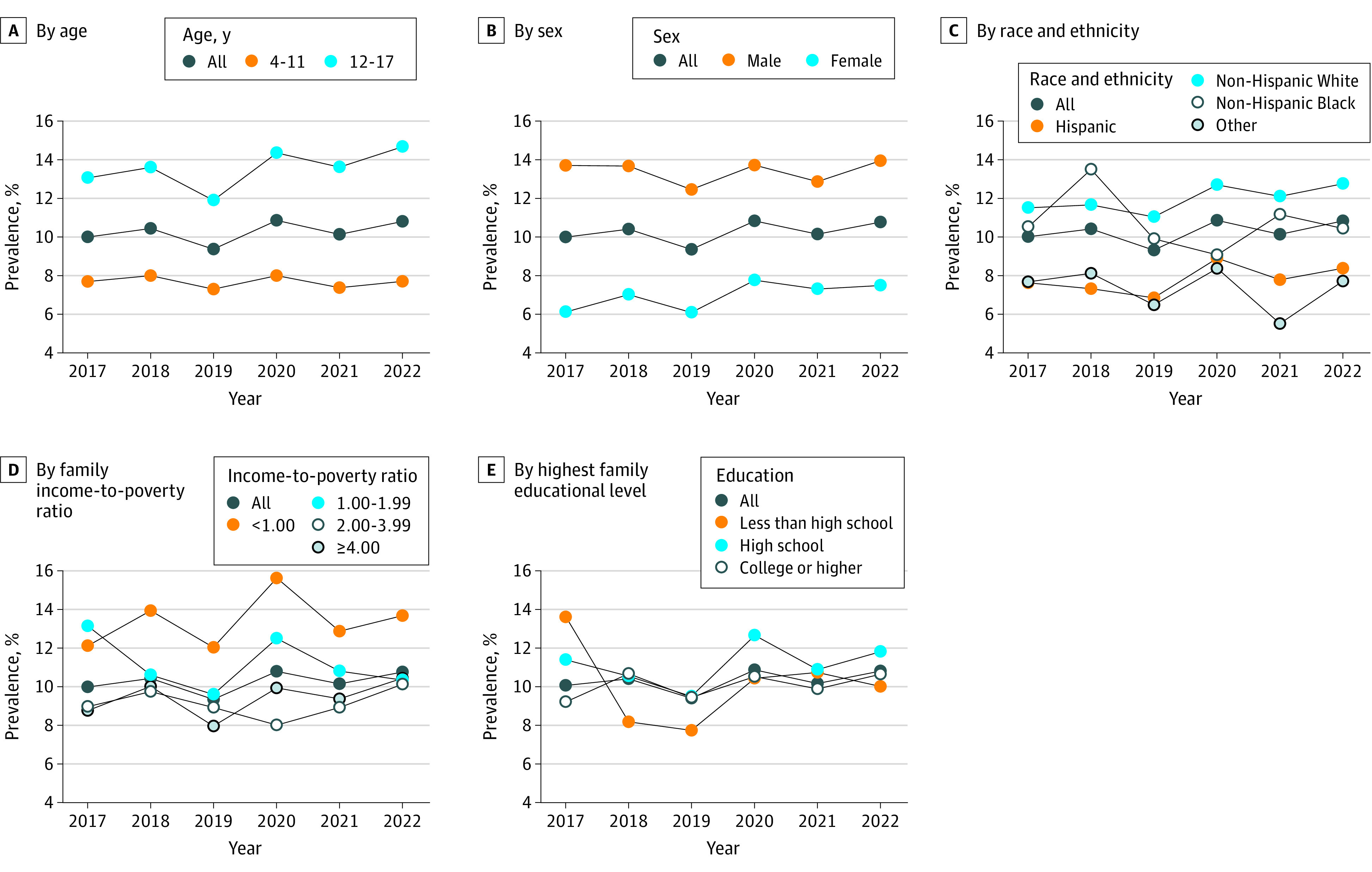
Trends in Prevalence of Attention-Deficit Hyperactivity Disorder (ADHD), 2017-2022 Prevalence estimates were weighted and are among children and adolescents aged 4 to 17 years. The weighted prevalence of ADHD was 10.00% (95% CI, 9.11%-10.90%) in 2017, 10.41% (95% CI, 9.42%-11.39%) in 2018, 9.34% (95% CI, 8.51%-10.16%) in 2019, 10.83% (95% CI, 9.61%-12.04%) in 2020, 10.14% (95% CI, 9.33%-10.96%) in 2021, and 10.80% (95% CI, 9.83%-11.76%) in 2022. *P* for trend was calculated using a weighted logistic regression model, which included survey year as a continuous variable and was adjusted for age (4-17 years: *P* = .31; 6-11 years: *P* = .79; 12-17 years: *P* = .07), sex (female: *P* = .04; male: *P* = .95), race and ethnicity (Hispanic: *P* = .52; non-Hispanic Black: *P* = .08; non-Hispanic White: *P* = .55; other: *P* = .46), family income-to-poverty ratio (<1.00: *P* = .61; 1.00-1.99: *P* = .30; 2.00-3.99: *P* = .35; ≥4.00: *P* = .11), and highest educational level of family members (<high school: *P* = .74; high school: *P* = .50; ≥college: *P* = .33). Other races and ethnicities included non-Hispanic American Indian or Alaska Native (individual only), non-Hispanic American Indian or Alaska Native and any other group, non-Hispanic Asian (individual only), and other single and multiple races or declined to respond, no response, or unknown. The family income-to-poverty ratio is the total family income divided by the poverty threshold.

## Discussion

Based on US national representative data, the estimated ADHD prevalence was 10.08% to 10.47% among children and adolescents aged 4 to 17 years from 2017 to 2022, which was similar to the prevalence from the NHIS in 2015 to 2016 (10.20%).^2^ No significant annual change in the prevalence of ADHD was found from 2017 to 2022. Notably, the estimated prevalence of ADHD among individuals in the US in this study was higher than worldwide estimates (5.3%) in earlier years (1978-2005).^6^ The prevalence of ADHD differed significantly by age, sex, race and ethnicity, and family income-to-poverty ratio, consistent with previous study findings.^2,3^

This study has some limitations. First, information on ADHD provided by parents may lead to misreporting and recall bias. Second, the NHIS underwent a major redesign in 2019, which may affect comparability with prior years, and the COVID-19 pandemic affected data collection in 2020, which may also affect survey estimates. Given that the estimated ADHD prevalence was still high, further investigation is warranted to assess potentially modifiable risk factors and provide adequate resources for treatment of individuals with ADHD in the future.

## Notes

Supplement.
Data Sharing Statement
