# Supplementary material for: Prevalence and Trends in Diagnosed ADHD Among US Children and Adolescents, 2017-2022
Source: JAMA Netw Open. 2023 Oct 4;6(10):e2336872. doi: 10.1001/jamanetworkopen.2023.36872 (PMC10551769; doi:10.1001/jamanetworkopen.2023.36872)
Supplement: Supplement. — Data Sharing Statement [file jamanetwopen-e2336872-s001.pdf]

Data Sharing Statement

Li. Prevalence and Trends in Diagnosed ADHDttention Deficit/ Hyperactivity Disorder Among US Children and Adolescents, From 2017- to 2022. *JAMA Netw Open*. Published online October 4, 2023. doi:10.1001/jamanetworkopen.2023.36872

Data
Data available: No
